# Spatiotemporal Changes of Chemical Fertilizer Application and Its Environmental Risks in China from 2000 to 2019

**DOI:** 10.3390/ijerph182211911

**Published:** 2021-11-12

**Authors:** Yuanzhi Guo, Jieyong Wang

**Affiliations:** 1 Institute of Geographic Sciences and Natural Resources Research, Chinese Academy of Sciences, Beijing 100101, China; guoyz.16b@igsnrr.ac.cn; 2 Key Laboratory of Regional Sustainable Development Modeling, Chinese Academy of Sciences, Beijing 100101, China

**Keywords:** chemical fertilizer application, environmental risks, agricultural production, food security, high-quality development, rural revitalization

## Abstract

Chemical fertilizers are important inputs in agricultural production. They not only increase crop yield but also bring many negative effects, such as agricultural non-point source pollution. Therefore, a scientific understanding of the regional differences in chemical fertilizer application and its environmental risks is of significance to promote China’s agricultural development. In this study, we analyzed the spatiotemporal pattern of chemical fertilizer application intensity (CFAI) in China since 2000, evaluated the environmental risks of provincial CFAI, and investigated the internal mechanism behind them. The results showed that the total amount and intensity of chemical fertilizer application in China from 2000 to 2019 presented a trend of increasing first and then decreasing. In 2000 and 2019, provincial CFAI in eastern China was generally higher than that in central and western China, and the environmental risks of provincial CFAI were spatially characterized by “high in the north and low in the south”. Factors such as poor soil conditions, unreasonable farming structure and backward fertilization methods are the main reasons for the continuous increase in the total amount and intensity of chemical fertilizer application, while the construction of ecological civilization and the transformation of society and economy are the main reasons for their decline. Finally, measures such as targeted fertilization, adjusting the use structure of chemical fertilizers, improving fertilization methods and replacing chemical fertilizers with organic fertilizers are proposed to promote the quantity reduction and efficiency increase of chemical fertilizer application in China.

## 1. Introduction

Agriculture is not only the main source of human food and clothing but also the foundation supporting economic development and social progress [1,2]. Before the industrial revolution, agricultural development mainly depended on the self-recovery of soil fertility or the increase of soil organic matter through returning animal manure and straw to the field [3]. As a result, the development of agriculture was slow and the scale was small. By the middle of the 19th century, the emergence of chemical fertilizers opened a channel to provide nutrients for crop growth from outside the agricultural system, expanded the contents of material flow and energy cycle in agricultural system, and greatly promoted the development of agricultural production [4]. Through balancing nutrient composition and improving soil fertility, the application of chemical fertilizers effectively ensures the stable and high yield of grain, which makes great contributions to human survival and development. According to the existing research, the contribution rate of chemical fertilizers to the growth of food production in various countries is generally between 30% and 60% [5,6,7].

China is the most populous developing country in the world [8,9]. The long-standing problem of insufficient food and clothing makes the Chinese government attach great importance to agricultural development and continue to strengthen financial and policy supports to improve grain production capacity and ensure national food security [10,11,12]. After nearly 70 years of efforts, China’s grain yield per unit area has increased from 1029 kg/ha in 1949 to 6272 kg/ha in 2019, and the per capita output of grain has correspondingly increased from 209 kg/person to 475 kg/person [13], feeding about 22% of the world’s population with just 7% of the world’s farmland [14,15,16,17,18]. In terms of the driving force behind it, technological progress, especially the development of chemical fertilizer technology, has played a vital role [19,20,21]. Since 2006, China has been the biggest producer and consumer of chemical fertilizers in the world, producing more than a quarter of the world chemical fertilizers and consuming more than 30% every year.

As one of the important components of the green revolution [22], chemical fertilizers are the “food” of crop growth and the necessary means of production for agriculture. Thus, they have played a critical role in aspects such as ensuring national food security and guaranteeing social stability [21]. However, because of the traditional agricultural production mode and unreasonable fertilization methods, the overuse of chemical fertilizers is widespread in China’s agricultural production [23,24], and has been one of the main sources of agricultural non-point source pollution [25,26,27]. With the construction of ecological civilization and the enhancement of people’s ecological awareness, more and more attention has been paid to resource and environmental problems such as water pollution, air pollution and land degradation, which are caused by the excessive use of chemical fertilizers [16,19,25,28,29,30]. As a result, how to deal with these problems has become an important target of the government policies and measures [31]. Due to the lack of scientific understanding of soil nutrients, the unreasonable chemical fertilizer application structure is also common in agricultural production [32], especially the prominent problems such as attaching importance to chemical fertilizers and neglecting organic fertilizers, attaching importance to nitrogenous fertilizers and neglecting phosphate and potash fertilizers and attaching importance to major elements and neglecting trace elements, all of which have seriously hindered crop growth [20,33]. In general, the unreasonable and unscientific problems in China’s chemical fertilizer application not only damage basic soil fertility and increase the cost of grain production, but also affect the quantity and quality of agricultural products and threaten national food security.

Currently, China’s socialist modernization has entered a new stage of high-quality development, and the focus of rural development has shifted to rural revitalization [9,34]. In this context, the Chinese government actively promotes the construction of ecological civilization to optimize human–earth relationship and achieve the sustainable development of agricultural system [35,36]. These new situations inherently require promoting the quantity reduction and efficiency improvement of agricultural inputs, especially the chemical fertilizers, to realize the green and high-quality development of agriculture. China is a country with vast territory and significant regional differences [37]. A scientific understanding of regional differences in chemical fertilizer application and its environmental risks is of great significance to guide the rational application of chemical fertilizers and the transformation and upgrading of agriculture in different areas. Employing a dataset of chemical fertilizer application from 2000 to 2019, this study analyzes the spatiotemporal pattern of chemical fertilizer application intensity (CFAI) in China, measures provincial CFAI safety thresholds according to local soil conditions, and then reveals the environmental risks of chemical fertilizer application in different provinces. Ultimately, the driving mechanism behind the spatial inequality of chemical fertilizer application as well as the measures for agricultural sustainable development are discussed. These findings will contribute to the implementation of new development concepts, promote the construction of resource-saving and environment-friendly industrial system in rural China, and finally realize the modernization of agriculture and rural areas.

## 2. Materials and Methods

### 2.1. Chemical Fertilizer Application Intensity

CFAI is a concept which reflects the consumption of chemical fertilizers per unit area of land. It has two statistic calibers of cultivated area and sown area [38]. Based on relevant studies [38,39], the latter is employed in this study to investigate the regional pattern of China’s CFAI and its environmental risks, and the formula is given as follows:

(1) CFAI=CCF/SAC

where CCF denotes the consumption of chemical fertilizers, which refers to the quantity of chemical fertilizers applied in agricultural production in the year, including nitrogenous fertilizers, phosphate fertilizers, potash fertilizers, and compound fertilizers. For the convenience of comparison, CCF is calculated in terms of the volume of effective components by converting the gross weight of respective fertilizers into weight containing effective components, e.g., nitrogen content in nitrogenous fertilizers, phosphorous pentoxide contents in phosphate fertilizers, and potassium oxide contents in potash fertilizers. SAC denotes the total sown area of crops, which refers to the area of all land sown or transplanted with crops that are harvested within the calendar year. All crops harvested within the year are counted as sown area, regardless of being sown in the current year or the previous year, and crops that are sown this year but harvested in the coming year are excluded.

### 2.2. Environmental Risk Assessment

The concept of risk assessment generally has a long history [40], but environmental risk assessment (ERA), as a scientific field, originated only in the early 1970s [41]. An ERA is a process used to evaluate the quantitative and qualitative characteristics of environment that may be impacted due to exposure to one or more environmental stressors, such as chemicals, disease, invasive species, and climate change [42,43]. The environmental risks of CFAI are the possibility of ecological damage and environmental pollution caused by chemical fertilizer application in the process of agricultural production, which has the characteristic of being non-sudden. Here, the ERA of CFAI is calculated as follows:

(2) $$R_{i}=F_{i}/\left(F_{i}+T_{i}\right)$$

where *R_i_* is the environmental risk index of region *i*, *F_i_* is the CFAI of region *i*, and *T_i_* is the environmental safety threshold of CFAI of region *i*. According to the calculation of environmental risks, the value of *R_i_* varies from 0 to 1. When *R_i_* is 0.50, it means that *F_i_* and *T_i_* are equal, which is the critical point of environmental safety; when *R_i_* approaches 1, it means that *F_i_* greatly exceeds *T_i_*, i.e., there are extremely serious environmental risks in chemical fertilizer application; while *R_i_* approaching 0 means that *F_i_* is much lower than *T_i_*. According to the multiple of *F_i_* to *T_i_*, the environmental risks are divided into five types: safe, low risk, moderate risk, high risk and serious risk (Table 1).

### 2.3. Data Source and Processing

The basic geographic data used in this study come from the Resource and Environment Science and Data Center of Chinese Academy of Sciences (https://www.resdc.cn/) (accessed on 5 September 2021). Data on sown area of farm crops and chemical fertilizer application are obtained from China Statistical Yearbook. Data on the grade of cultivated land are collected from the Ministry of Natural Resources of the PRC (http://www.mnr.gov.cn/) (accessed on 5 September 2021). According to the research design, Hong Kong, Macao, and Taiwan are excluded in the analysis. As a result, a total of 31 provincial-level administrative units are obtained to investigate the regional differences of CFAI and their environmental risks in China.

## 3. Results

### 3.1. Evolution of Chemical Fertilizer Application in China

**Figure 1:**
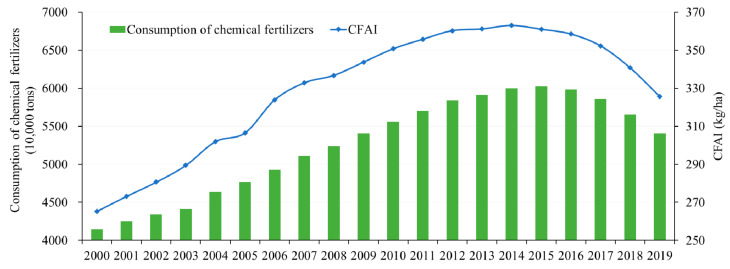
Evolution of chemical fertilizer application in China from 2000 to 2019.

In 2000, the total amount of chemical fertilizers applied in China’s agricultural production was 41.46 million tons, and increased to 54.04 million tons in 2019, with an average annual growth rate of 1.40%. Accordingly, China’s CFAI increased from 265.28 kg/ha to 325.65 kg/ha, and the average annual growth rate was 1.08%. Specifically, the consumption of chemical fertilizers maintained a stable growth trend before 2015, and the average annual growth rate during this period reached 2.52%. Then, as the Ministry of Agriculture and Rural Affairs of the PRC issued the action plan for zero growth of chemical fertilizer application by 2020 in February 2015 to promote the green development of agriculture, the total amount of chemical fertilizer application in China decreased at an average annual rate of 2.67% in 2015–2019. The evolution of CFAI also showed a trend of increasing first and then decreasing, but the turning year was advanced to 2014, and the average annual growth rate of the two stages changed from 2.26% to −2.15% (Figure 1).

**Figure 2:**
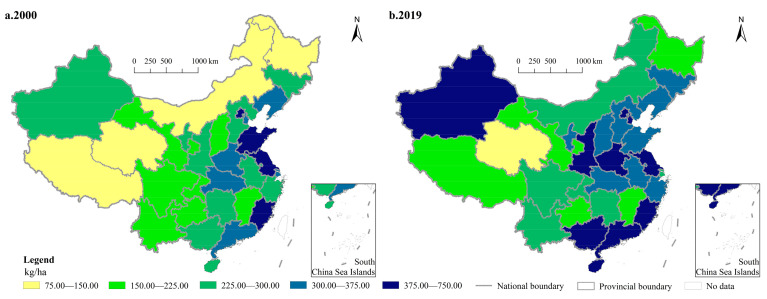
Spatial pattern of China’s provincial CFAI in 2000 (**a**) and 2019 (**b**).

In terms of the provincial CFAI, CFAI in Tibet was the lowest in 2000, only 108.18 kg/ha, followed by Inner Mongolia (126.47 kg/ha), Qinghai (130.03 kg/ha) and Heilongjiang (130.34 kg/ha). These provinces were the only four provinces with a CFAI lower than 150 kg/ha. The province with the highest CFAI was Fujian, followed by Jiangsu, Beijing and Shandong, where the value of CFAI was all greater than 375 kg/ha (Figure 2a). After nearly two decades of development, provincial CFAI has developed rapidly. In 2019, Qinghai was the province with the smallest CFAI, which was just 112.01 kg/ha and the only province with a CFAI lower than 150 kg/ha in the whole country. On the other end of the spectrum were three eastern provinces, Beijing, Hainan, and Fujian, where CFAI was 699.77 kg/ha, 684.71 kg/ha and 664.67 kg/ha, respectively; CFAIs of these three provinces were much greater than those of other provinces (Figure 2b). Spatially, provincial CFAI showed an obvious east-central-west gradient differentiation in 2000 and 2019, that is, provincial CFAI in the eastern coastal region was significantly greater than that in the central region, and provincial CFAI in the western region was generally small.

The analysis of the changes of provincial CFAI from 2000 to 2019 showed that CFAI decreased in only five provinces, namely, Shanghai, Qinghai, Jiangsu, Shandong and Guizhou, with a decrease of 22.59%, 13.86%, 8.94%, 4.76% and 0.02%, respectively. Among the provinces with an increase of CFAI, there were eleven provinces with an increase greater than 50%, and the greatest was Hainan (135.87%), followed by Inner Mongolia (94.36%), Xinjiang (78.93%) and Beijing (78.77%); only CFAI in Liaoning, Hubei and Sichuan increased by less than 10%, and the value was 9.43%, 7.56% and 3.89%, respectively. In general, the changes of provincial CFAI showed a characteristic of being small in traditional agricultural areas and large in non-traditional agricultural areas.

### 3.2. Environmental Risks of Chemical Fertilizer Application in China

**Figure 3:**
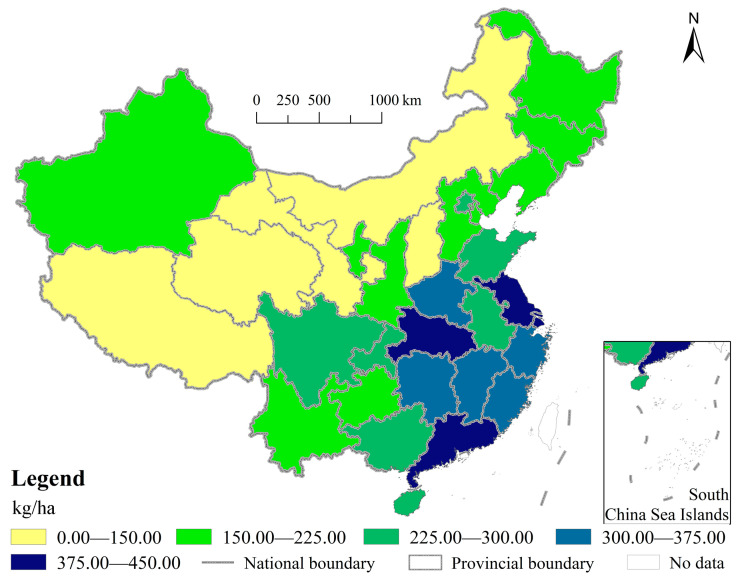
Spatial pattern of provincial environmental safety upper limits of CFAI in China.

To measure the environmental risks of chemical fertilizer application in different provinces, it is necessary to first determine the provincial safety upper limit of CFAI. According to existing research, the internationally recognized ceiling for safe CFAI is 225 kg/ha [44,45]. Here, we also set this value as the safety upper limit of CFAI in China, that is, the ceiling of CFAI corresponding to 9.96-grade cultivated land is 225 kg/ha. The calculation of China’s CFAI over the years shows that CFAI in China has exceeded the safety upper limit since 1995, and has been running at a high level for a long time [13]. Because of the regional inequality in cultivated land grades derived from the spatial heterogeneity of natural and human conditions [37,46], the provincial safety upper limit of CFAI is also different. In line with the safety upper limit of CFAI, which is corresponding to the national average grade of cultivated land, the cultivated land grades of different provinces are employed to calculate their safety upper limits of CFAI. As shown in Figure 3, the environmental safety threshold of chemical fertilizer application in each province shows a significant gradient descent pattern from the southeast coastal areas to the northwest inland areas. Specifically, there are sixteen provinces with a safety upper limit of CFAI greater than 225 kg/ha, mainly distributed in the eastern coastal region and the central region with good natural conditions, such as abundant water and good soil. Among them, there are four provinces with a safety upper limit greater than 375 kg/ha, including Hubei (428.42 kg/ha), Guangdong (424.98 kg/ha), Shanghai (411.64 kg/ha) and Jiangsu (399.27 kg/ha). Jiangxi and Henan are also close to that level. It is noted that the provinces with a safety upper limit less than 225 kg/ha mainly distribute in the western and northeast regions, including Inner Mongolia, Gansu, Qinghai, Shanxi, and Tibet, where the upper safety limits are all less than 150 kg/ha.

**Figure 4:**
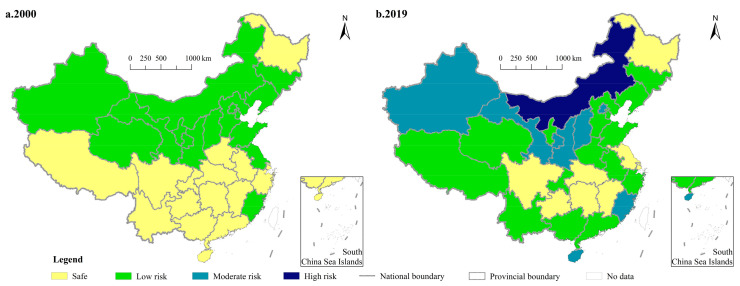
Spatial pattern of the environmental risks of China’s CFAI in 2000 (**a**) and 2019 (**b**).

Due to the relative stability of natural conditions [47], we assume that the cultivated land grade of each province is the same in 2000 and 2019. Based on provincial CFAI and their environmental safety upper limits, formula (2) is employed to calculate the environmental risks of chemical fertilizer application, and ArcGIS 10.4 is used for spatial visualization (Figure 4). In 2000, there were only two types of environmental risks for provincial CFAI, namely, safety and low risk. The former included sixteen provinces, mainly distributed in the south, of which the province with the lowest risk was Jiangxi (0.36), followed by Hubei (0.43) and Hunan (0.43). The other provinces belonged to the latter, and the province with the highest risk level was Gansu (0.66), with Shaanxi (0.64) and Liaoning (0.64) being close to that level. In 2019, there were only eight provinces whose environmental risk type was safety, mainly distributed in the middle and upper reaches of the Yangtze River. Among them, the province with the lowest environmental risk was Jiangxi (0.38), followed by Shanghai (0.41) and Hubei (0.45), and the other five provinces were on the edge of the safety line. The number of provinces with a risk type of moderate risk was seven; except for Hainan, Fujian and Beijing in the east, the rest were mainly distributed in the northwest. Only the risk type of Inner Mongolia was high risk.

In terms of the changes of environmental risks of CFAI during the period of 2000–2019, there were five provinces with a decreased value of environmental risks, namely Shanghai, Qinghai, Jiangsu, Shandong and Guizhou, and the decrease was 13.31%, 6.36%, 4.55%, 2.15% and 0.01%, respectively. Among the provinces with an increased value of environmental risks, there were eight provinces with an increase greater than 20%, of which the highest was Hainan (41.06%), followed by Tibet (27.87%) and Guangxi (27.39%). The analysis of the changes of environmental risk types from 2000 to 2019 showed that there were eight provinces changing from safe to low risk and one province changing from safe to moderate risk; there was one province changing from low risk to safe, six provinces changing from low risk to moderate risk, and one province changing from low risk to high risk; there were seven provinces whose risk types remained safety, and the number of provinces whose risk type has always been low risk was also 7 (Table 2).

## 4. Discussion

### 4.1. Understanding the Chemical Fertilizer Application in China

Through solving the problem that soil nutrients cannot meet the needs of crop growth, chemical fertilizers effectively promote agricultural development, thus ensuring national food security [19]. Wang et al. pointed out that 40% of the increase in China’s agricultural production during the period of 1986–1990 came from the increase of chemical fertilizer application [48]. However, the growth rate of chemical fertilizer application in this process has far exceeded the growth rate of grain output, resulting in a serious problem of excessive and inefficient application of chemical fertilizers, which become an important challenge restricting the sustainable development of agriculture in China [16,49]. According to statistics from the Ministry of Agriculture and Rural Affairs of the PRC, the comprehensive utilization rate of chemical fertilizers for rice, wheat, and corn in 2020 was only 40.2%, which was far lower than that of 60%~70% in developed countries. As the products of technological progress, chemical fertilizers are essentially harmless. The problem related to chemical fertilizers in agricultural production is not the problem of chemical fertilizers itself but stems from the unreasonable and unscientific use of chemical fertilizers [50], including unreasonable farming structure and backward fertilization method, as well as insufficient scientific and technological guidance.

**Figure 5:**
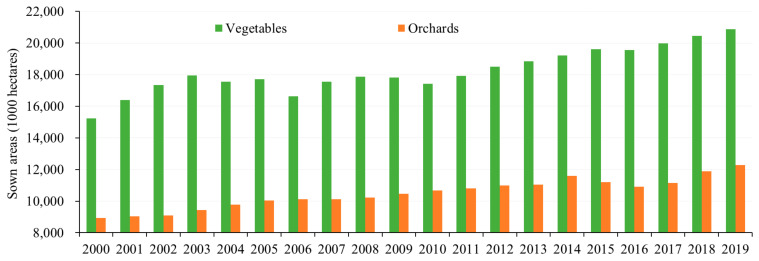
Changes of the sown areas of vegetables and orchards in China from 2000 to 2019.

First, China’s per capita cultivated land is small, less than 0.10 ha/person, and the basic fertility of cultivated land is low, with medium and low yield cultivated land accounting for nearly 65%. To ensure national food security, increasing the use of chemical fertilizers has become an important choice in agricultural production [19,51]. However, due to the imperfect fertilizer management system, the massive application of chemical fertilizers has caused many resources and environmental problems, such as soil degradation and the decline of land productivity, which further increases the use of chemical fertilizers, forming a vicious circle [52]. Meanwhile, the low-quality cultivated land is poor in water and fertilizer conservation, reducing the utilization efficiency of chemical fertilizers. Second, the adjustment of farming structure boosts the increase of chemical fertilizer application. Guided by a market economy and consumption upgrading, more and more cultivated land in China is used to grow non-grain crops [8], such as fruits and vegetables (Figure 5), which have a higher demand for chemical fertilizers [53,54,55]. Due to the traditional fertilization habits and the lack of understanding of fertilizer performance, the phenomenon of excessive chemical fertilizer application is widespread in the process of cash crops production, especially in the eastern developed region [56]. Third, the dominant position of small-scale peasant economy in rural China makes the agricultural mechanization develop slowly. In this context, the traditional artificial fertilization is still dominant, such as spreading and surface application, which leads to the volatilization and leaching of chemical fertilizers and reduces the utilization rate of chemical fertilizers. Fourth, due to the urban–rural dual structure and urban-biased development strategy, problems such as imperfect agricultural technology service system and few technicians are common in rural China, which make farmers unable to get guidance from technicians in chemical fertilizer application. Fifth, with the rapid development of industrialization and urbanization, many rural working people flow to urban areas. This results in the aging and weakening of agricultural producers, and causes the substitution of modern production factors such as chemical fertilizer for labor force, which further increases CFAI [57]. Additionally, in the process of circulation, fertilizer dealers tend to increase the recommended application number of chemical fertilizers to obtain more benefits, resulting in excessive chemical fertilizer application [58].

After more than 30 years of reform and opening-up, China’s economy developed to the new normal stage in 2014, which requires optimizing the economic structure and realizing the transformation and upgrading of economic growth from factor-driven to innovation-driven [59,60]. Against this background, China’s agricultural development has gradually changed from extensive growth of scale–speed type to intensive growth of quality–efficiency type, constantly optimizing the structure of chemical fertilizer application and improving the efficiency of chemical fertilizer application. On the other hand, the report to the 18th National Congress of the Communist Party of China (CPC) proposed to vigorously promote the construction of ecological civilization and form resource-saving and environment-friendly spatial pattern, industrial structure, production and living mode to reverse the trend of ecological and environmental deterioration from the source [61]. To achieve this goal, the Chinese government has issued a series of policies and measures to promote the quantity reduction and efficiency improvement of agricultural inputs such as chemical fertilizers and pesticides, and paid attention to the comprehensive utilization of agricultural wastes, thus building a high-efficiency, low-carbon and green agriculture system. Moreover, the promotion of rural reform continues to help the rapid development of various new agricultural business entities [62], which promote the scale of agricultural production and improves the efficiency of chemical fertilizer application. Driven by the transformation of major national policies, the total amount and intensity of chemical fertilizer application in China has decreased steadily since the late 12th Five-Year Plan, and this trend will continue, thus boosting the high-quality development of agriculture and achieving the goal of agricultural and rural modernization.

### 4.2. Policy Implications for Agricultural Development

Since 2004, China’s grain production has achieved bumper harvests for 16 consecutive years. However, the grain supply and demand is still in a tight balance, and the food-security situation remains grim [8,63]. As people’s food demand changes from enough to high-quality and diversified, the grain consumed in the production of meat, eggs, milk and other food will continue to increase, which leads to rapid growth of grain consumption [64,65,66]. On the other hand, the restrictive factors such as fresh water resources and environmental carrying capacity have rigid constraints on grain production, and become increasingly prominent, which increases the difficulty of increasing grain production [67,68,69]. To ensure national food security and meet people’s needs for a better life, chemical fertilizers must continue to be used. In the foreseeable future, chemical fertilizers will continue to play an important role in China’s agricultural production, and more attention should be paid to scientific fertilization to maximize its socioeconomic benefits and avoid the adverse impacts of unreasonable and unscientific fertilization on resources and environment. Meanwhile, some targeted measures should be taken to establish and improve the chemical fertilizer application system to ensure production, save cost and increase efficiency according to local conditions.

First, it is necessary to promote targeted fertilization. According to soil conditions, crop yield potential and the requirements of comprehensive nutrient management, the fertilization quota standard per unit area of crop should be reasonably formulated in different regions to reduce blind fertilization. Second, more attention should be paid to adjust the use structure of chemical fertilizers. By optimizing the ratio of nitrogen, phosphorus and potassium fertilizers, the reasonable combination of major element and medium and trace element can be realized. Meanwhile, there is an urgent need to guide the optimization and upgrading of fertilizer products to meet the need of modern agriculture. Third, fertilization method needs to be improved. The technology of soil testing and formula fertilization (*Cetu Peifang Shifei*) should be popularized to improve farmers’ skills of scientific fertilization. The government also should promote suitable fertilization equipment and guide the changes of fertilization methods from surface application and spreading application to mechanical deep application, water and fertilizer integration and foliar spraying. Fourth, chemical fertilizers should be replaced by organic fertilizers. Through rational utilization of organic nutrient resources, organic fertilizers are used to replace part of chemical fertilizers, realizing the rational combination of organic and inorganic fertilizers. In addition, some guarantee mechanisms need to be established and improved to ensure the implementation of these measures for quantity reduction and efficiency increase of chemical fertilizers, including a working mechanism of up–down linkage and multiparty cooperation, a national fertilizer efficiency monitoring network, publicity and training of new business entities, and supporting policies such as finance and taxation.

### 4.3. Limitations and Future Research Prospects

The objects of farming include not only grain crops, such as rice and corn, but also cash crops, such as vegetables and fruits [8], and there are significant differences in nutrient elements required for the growth of different crops. As a result, their demands for nitrogen fertilizers, phosphorus fertilizers, potassium fertilizers, compound fertilizers and other types of chemical fertilizers are different. Here, we only discuss the overall situation of regional chemical fertilizer application, but lack of understanding of the use structure of chemical fertilizers, the regional differences of different types of chemical fertilizers and their environmental risks. Therefore, the chemical fertilizer application of different crops is worthy of further investigation. When measuring the environmental risks of provincial CFAI, the environmental safety threshold used in this study is a value calculated by international general standards. In fact, the environmental safety threshold is closely related to the nutrient situations of cultivated land [70]. Thus, it is necessary to further promote soil testing in future research and comprehensively determine whether chemical fertilizer application is excessive according to regional soil fertility, thus scientifically guiding the chemical fertilizer application in agricultural production and avoiding various resource and environmental problems caused by the overuse of chemical fertilizers. In addition, with the rapid development of society and the economy, greenhouse gas emissions dominated by carbon dioxide are posing a serious threat to the global climate and ecology [71,72]. In this context, China is actively committed to the implementation of the Paris Agreement, striving to reach the peak of carbon dioxide emissions by 2030 and achieve carbon neutralization by 2060. The process of chemical fertilizer production is an important carbon source, and chemical fertilizer application also produces a large amount of carbon dioxide [73,74]. Therefore, scientific discussion on the relationship between chemical fertilizer production/application and carbon emission and its internal mechanism is of great significance to achieve the goal of carbon emission reduction in China.

## 5. Conclusions

Currently, China’s agricultural development is in the critical stage of transformation and upgrading, and its driving force is changing from factor input to technological innovation [75]. Against this background, optimizing the structure and efficiency of agricultural inputs, such as chemical fertilizers, has become an important measure to promote agricultural development to a higher level in the new era. Affected by poor soil conditions, unreasonable farming structure and backward fertilization methods, the total amount and intensity of chemical fertilizer application in China have maintained an increasing trend for a long time. With the promotion of ecological civilization and rural reform, the total amount and intensity of chemical fertilizer application began to decline at the end of the 12th Five-Year Plan. Spatially, provincial CFAI in 2000 and 2019 showed a pattern of “high in the east and low in the west”, and the changes of non-traditional agricultural areas were more obvious than those of traditional agricultural areas. The analysis of the CFAI safety thresholds showed that the provinces with a high safety threshold were mainly distributed in the third terrain ladder, and those with a low safety threshold were mainly distributed in northwest China and Qinghai–Tibet Plateau. As a result, the environmental risks of provincial CFAI in 2000 and 2019 were all characterized by “high in the north and low in the south”. To solve excessive application of chemical fertilizers and its related problem in China, it is necessary to promote the quantity reduction and efficiency increase of chemical fertilizers through targeted fertilization, adjusting the use structure of chemical fertilizers, improving fertilization methods, and replacing chemical fertilizers with organic fertilizers, thus realizing agricultural green and high-quality development and supporting agricultural and rural modernization.

## Figures and Tables

**Table 1:** Classification of the environmental risks of chemical fertilizer application.

Classification	Type	Threshold	Criteria
Level Ⅰ	Safe	0.00 < *R_i_* ≤ 0.50	*F_i_* ≤ *T_i_*
Level Ⅱ	Low risk	0.50 < *R_i_* ≤ 0.67	*T_i_* < *F_i_* ≤ 2*T_i_*
Level Ⅲ	Moderate risk	0.67 < *R_i_* ≤ 0.75	2*T_i_* < *F_i_* ≤ 3*T_i_*
Level Ⅳ	High risk	0.75 < *R_i_* ≤ 0.80	3*T_i_* < *F_i_* ≤ 4*T_i_*
Level Ⅴ	Serious risk	0.80 < *R_i_* < 1.00	*F_i_* > 4*T_i_*

**Table 2:** Changes of the environmental risk type of provincial CFAI in China from 2000 to 2019.

		2019			
		Safe	Low Risk	Moderate Risk	High Risk
2000	Safe	Jiangxi, Shanghai, Hubei, Sichuan, Guizhou, Hunan, Heilongjiang	Anhui, Chongqing, Zhejiang, Guangdong, Tibet, Henan, Guangxi, Yunnan	Hainan	
	Low risk	Jiangsu	Qinghai, Shandong, Tianjin, Ningxia, Liaoning, Jilin, Hebei	Fujian, Shanxi, Gansu, Beijing, Xinjiang, Shaanxi	Inner Mongolia

## Data Availability

The associated dataset of the study is available upon request to the corresponding author.
